# Bayesian modeling reveals host genetics associated with rumen microbiota jointly influence methane emission in dairy cows

**DOI:** 10.1038/s41396-020-0663-x

**Published:** 2020-05-04

**Authors:** Qianqian Zhang, Gareth Difford, Goutam Sahana, Peter Løvendahl, Jan Lassen, Mogens Sandø Lund, Bernt Guldbrandtsen, Luc Janss

**Affiliations:** 1 Department of Molecular Biology and Genetics, Center for Quantitative Genetics and Genomics, Aarhus University, Aarhus, Denmark; 2 Present Address: Bioinformatics Research Centre, Aarhus University, Aarhus, Denmark; 3 Present Address: Nofima, Norwegian Institute of Food Fisheries and Aquaculture Research, N-1430 Ås, Tromsø, Norway

**Keywords:** Microbial genetics, Microbial genetics

## Abstract

Reducing methane emissions from livestock production is of great importance for the sustainable management of the Earth’s environment. Rumen microbiota play an important role in producing biogenic methane. However, knowledge of how host genetics influences variation in ruminal microbiota and their joint effects on methane emission is limited. We analyzed data from 750 dairy cows, using a Bayesian model to simultaneously assess the impact of host genetics and microbiota on host methane emission. We estimated that host genetics and microbiota explained 24% and 7%, respectively, of variation in host methane levels. In this Bayesian model, one bacterial genus explained up to 1.6% of the total microbiota variance. Further analysis was performed by a mixed linear model to estimate variance explained by host genomics in abundances of microbial genera and operational taxonomic units (OTU). Highest estimates were observed for a bacterial OTU with 33%, for an archaeal OTU with 26%, and for a microbial genus with 41% heritability. However, after multiple testing correction for the number of genera and OTUs modeled, none of the effects remained significant. We also used a mixed linear model to test effects of individual host genetic markers on microbial genera and OTUs. In this analysis, genetic markers inside host genes *ABS4* and *DNAJC10* were found associated with microbiota composition. We show that a Bayesian model can be utilized to model complex structure and relationship between microbiota simultaneously and their interaction with host genetics on methane emission. The host genome explains a significant fraction of between-individual variation in microbial abundance. Individual microbial taxonomic groups each only explain a small amount of variation in methane emissions. The identification of genes and genetic markers suggests that it is possible to design strategies for breeding cows with desired microbiota composition associated with phenotypes.

## Introduction

Mitigation of greenhouse gas emissions has become a major research objective due to global climate change [1–3]. With the development of tools for the measurement of methane emission in the livestock sector, the amount of greenhouse gas such as methane emission from cows can be measured on a large scale [4, 5]. Global livestock production is an important source of greenhouse gasses. For example, livestock accounts for 10–12% of CO_2_, and 5% of CH_4_ of total anthropogenic greenhouse gas emissions [6, 7]. Growing human populations and incomes are fueling demand for animal products, which result in more greenhouse gas emission from livestock production. Therefore, reduction of methane emission from livestock production is critical to address the global climate problems. Besides, methane emission constitutes a loss of dietary energy for the cow. Thus, reducing methane emissions maybe ties to improving feed efficiency.

Among livestock species, ruminants such as cattle have the ability to digest rough fiber feeds like grasses, thanks to the action of symbiotic ruminal microbes. This process leads to production of methane as a byproduct [8]. Studies have shown that the amount of methane emissions from cow rumen is largely determined by the composition of its ruminal microbiota, feed intake and diet composition, physiology and genome [9–13]. Methane is produced in ruminants when anaerobic archaeal microorganisms convert H_2_ and CO_2_ to CH_4_ [14]. Therefore, among different microbial groups, archaea play an important role in methane production. The link between microbiota and methane emission is well known. Recent studies have found that host genetics influences methane emission [15–18]. Therefore, it might be possible to identify both properties of the microbiota and host genetic markers as biomarkers to select cows with low methane emissions.

However, until now, there has been little success in identifying robust biomarkers useful to reduce methane emission in ruminants like cows. This is due to insufficient data or a lack of statistical models. None the less, Roehe et al. [16] identified 20 microbial genes associated with methane yield using ~8 samples. Furthermore, using 16S rRNA ribotyping on 68 postmortem rumen samples, they also found evidence that the archaea:bacteria ratio and methane yield are similarly influenced by Sire [16]. Their study opened up the possibility of selecting cows with lower methane emission based on the archaea:bacteria ratio in the rumen. Breeding for less methane emitting cows through genetic selection is a sustainable approach to reduce climate impact. To this end, we need more knowledge about how host genetics and microbiota affect methane emission [19, 20]. Larger sample sizes and new statistical models will improve our knowledge on how host genetics and which host genes affect microbiota composition, and which taxonomic groups in the microbiota affect methane emission. Accurate knowledge on the contributions of host genetics and microbiota to variation in methane emission will also aid in understanding biological underlying variation in methane emission. Such knowledge can be utilized to design a strategy to reduce methane emissions from the cow rumen.

Microbiota data are high dimensional and zero inflated [21, 22]. Correlation patterns between microbiota and environmental covariates are usually studied by various regression models [23–25]. Roehe et al. and Ross et al. were the first to model the influence of host genetics on methane emission through ruminal microbial community in cows [16, 26]. Ross et al. and Difford et al. have proposed using a similarity matrix built from microbiota 16S rRNA abundance and shotgun metagenomic contig data [15, 26]. This matrix was fitted as a covariance structure for a random effect in a linear mixed model [15, 26]. In their model, all components of the microbiota are implicitly assumed to contribute equally to genetic variance. A Bayesian mixture model is able to derive the posterior distribution of the effect of each specific microbiota under a mixture of prior distributions [27]. In this way, the effects of microbiota are jointly modeled, and therefore, the complex structure and interplay between microbiota can be modeled.

In this study, we used both Bayesian model and mixed linear model approaches, methane emission data from 750 cows together with their SNP chip genotypes as genetic markers, and measures of microbial abundance. We modeled joint effects of host genetics and all microbial abundances on methane emission, as well as host heritability and SNP effects on abundances in individual bacterial genera. We hypothesize that (1) a Bayesian model that allows for joint effects of host and all bacterial genera can be used to explore the variation explained by microbiota data in methane emission; (2) host genetics and genes in host genomes influence the microbiota composition, (3) the abundance of different microbiota influence methane emission; (4) both host genetics and microbiota variation contribute to the variation in methane emission.

## Material and methods

All handling of animals was conducted according to “Metagenomics in Dairy Cows” protocol. The protocol and study were approved by The Animal Experiments Inspectorate, Danish Veterinary and Food Administration, Ministry of Environment and Food of Denmark (Approval number 2016-15-0201-00959).

### Phenotype and genotype collection

Data of methane emissions from 750 Holstein cows were collected, see [15]. Concentrations of CH_4_ and CO_2_ were measured spectroscopically in the breath of individual cows during automated milking in voluntary milking stations. Mean gas concentrations were corrected for systematic effects such as diurnal variation and day-to-day differences following [15]. Total methane emissions were calculated as the measured CH_4_ to CO_2_ ratio (l/day) and converted CO_2_ (g/d) using CH_4_ density times the predicted CO_2_ emission from the converted cow heat production units to CO_2_ production [4].

Out of 750 cows, 691 cows were genotyped using Illumina BovineSNP50 BeadChip (50k) versions 2 (Illumina Inc., San Diego, CA). SNPs were removed from further analysis if they met any of these criteria: no known chromosomal location according to Illumina’s maps [5], non-autosomal locations, call rates <99% for individuals or <98% for SNPs, deviation from Hardy–Weinberg proportions (*p* < 10^−^^8^), or minor allele frequency <0.02. In total, 39,034 autosomal SNPs remained for the analyses. Pedigree records for these 750 cows traced as far back as far as 1926.

### 16S rRNA sequencing of microbiota, sequence processing, and OTU table construction

Rumen liquid fractions were collected individually from the rumen of the sampled cows by oral insertion of the rumen floral scoop [28]. For details on rumen liquid fraction collection, see [15]. DNA extraction of rumen liquid fraction was performed using Qiagen QIAamp toolkit according to manufacturer’s instructions. DNA library construction and sequencing of DNA were performed by Eurofins Genomics. 16S rRNA sequencing was conducted using the universal bacterial 16S gene primers (V1–V3) and universal archaeal 16S rRNA gene primers (V4–V6) [13, 28]. In total, 96 libraries were sequenced using 250 bp paired-end short read sequencing. Half the samples were sequenced on the Illumina MiSeq platform, the other half on the Illumina HiSeq platform. For details of 16S rRNA sequencing of the microbiota, see [15]. The sequence reads of 16S rDNA of bacterial and archaeal were processed under quality control, see [15]. They were clustered into operational taxonomic units (OTUs) using the LotuS pipeline [29]. For details on sequence processing and quality control, see [15]. Consensus sequences were derived for each OTU. A phylogenetic tree of the consensus sequences was constructed. Further, taxonomies were assigned to OTUs using the RDP classifier with a confidence level of 0.8 using the greengenes reference database (gg_13_8_otus) [30]. We retained the OTUs classified as k_Bacteria and k_Archaea for the bacterial primer set and the archaeal primer set, respectively.

### Statistical models

#### Variance explained by microbiota using Bayesian four-component mixture model

We used a Bayesian model to jointly model effects of host genetics and all microbiota on host methane emission. This model is a generalized version of that used by BayesR [27]. Like BayesR, we assign a four-component mixture prior to effect sizes (i.e., each OTU is assumed to have a tiny, small, medium, or large effect on methane emissions). However, we, in addition, include a polygenic term, in order to capture the contribution of host genetics. We implemented the model in Bayz (see http://www.bayz.biz); an R version of Bayz is freely available at github.com/MarniTausen/BayzR/ (released under a GPL license) [35–37].

The conditional distribution of the data was taken as:

$${\mathbf{y}}|{\mathbf{\gamma }},{\mathbf{u}},{\mathbf{\beta }},\sigma _{\rm{e}}^2\sim {\mathbf{N}}({\mathbf{X\gamma }} + {\mathbf{Zu}} + {\mathbf{M\beta }},{\mathbf{I}}\sigma _{\rm{e}}^2),$$

where **y** was a vector of estimated methane production (l/day). **X** was a matrix of fixed covariates including an intercept, dummy covariates for herd (six levels) and parity (four levels), a covariate for days in milk (range: 1–350), and covariates to fit a Wilmink function on days in milk accounting for nonlinearity in early lactation [31], and **γ** is a vector of associated effects. **Z** was an incidence matrix relating phenotypes to the corresponding polygenic effects **u**. **M** was an *n* × *m* matrix of the counts of the *m* tested OTUs and *n* was the number of individuals. To construct **M**, first, OTUs which were not present in at least 50% of the cows were removed. The element *m*_*ij*_ was then added a small constant (0.001) and natural log transformed and then standardized for the *j*’th OTU in individual *i* with mean of 0 and variance of 1. In order to account for the compositional nature of **M**, a separate centered log-ratio (CLR) transformation was conducted as per [32] using the CoDaSeq package [33, 34]. Both methods of transforming data were run separately. **β** was the vector of associated effects of the *m* OTUs within sequencing instrument as described in [14]. Residuals were assumed independent and identically distributed with residual variance $\sigma _{\rm{e}}^2$.

The prior distributions of the model parameters were as follows. The effects in **γ** were assumed independently distributed, each with an unbounded uniform prior, which can be written as:

$$\gamma _{\rm{i}} \propto {\rm{constant}}.$$

The vector of polygenic effects **u** had a multivariate Normal prior:

$$u\sim N(0,{\mathbf{A}}\sigma _u^2),$$

where **A** is the known pedigree-based additive genetic relationship matrix, and $\sigma _u^2$ is an unknown polygenic variance, which was estimated with an unbounded uniform prior:

$$\sigma _{\rm{u}}^2 \propto {\rm{constant}}.$$

The vector of effects of OTUs **β** was modeled to have a four-component mixture distribution. To facilitate fitting of this mixture, indicator variables *z*_*i*_ were added in the model, where *z*_*i*_ has a categorical distribution *zi* ∈{1,2,3,4} to indicate the (unknown) assignment of the *i*'th OTU to one of the four components of the mixture. With this prior distribution for the *i*'th OTU effect is written as:

$$\begin{array}{l}{\upbeta}_{\mathrm{i}}\sim \left[ {z_{\rm{i}} = 1} \right]N({\mathbf{0}},{\mathrm{I}}{\it{\upsigma }}_1^2) + \left[ {z_{\mathrm{i}} = 2} \right]N(0,{\mathrm{I}}{\it{\upsigma }}_2^2) \\ \quad + \, \left[ {z_{\rm{i}} = 3} \right]N(0,{\mathrm{I}}\sigma _3^2) + \left[ {z_{\rm{i}} = 4} \right]N(0,{\mathrm{I}}\sigma _4^2)\end{array},$$

where [] are Iverson brackets and *β*_i_ are assumed independent. The variances in the components of the mixture have unbounded uniform priors, and are constrained so that $\sigma _1^2 = 10\sigma _2^2 = 100\sigma _3^2 = 1000\sigma _4^2$. The indicator variables *z*_i_ had a categorical distribution with unknown parameters *π*_k_ to indicate the probability for *z*_i_ = *k* as:

$$z_{\rm{i}}\sim {\mathrm{cat}}\left( {\pi _1,\pi _2,\pi _3,\pi _4} \right).$$

The *π*_k_ probabilities indicate the mixture proportions of the four-component mixture distribution. These mixture proportions were estimated from the data with a Dirichlet prior set as:

$$\left( {\pi _1,\pi _2,\pi _3,\pi _4} \right)\sim {\mathrm{Dir}}\left( {125,25,5,1} \right).$$

The rationale behind the four-component mixture is to apply different levels of shrinkage (regularization) to the OTU effects, with the model determining assignment to one of the four shrinkages (mixture components), and with priors such that most effects will be heavily shrunken inducing a sparse model. This is induced by the Dirichlet prior on the mixture proportions giving most prior counts to the first class, while the constraints on the variances induce highest shrinkage in the first class. Conversely, the last component of the mixture induces the mildest shrinkage, allowing *β*_i_ assigned to this group to remain relatively large, but a-priori this group is the smallest. The hyperparameters and priors were selected based on prior biological knowledge and previous literature [35–37]. In particular, the Dirichlet prior is strong enough to induce the desired sparse model, but weak enough to allow *π*_k_’s to be updated by information from the data, and the ratio of 1:1000 between strongest and weakest shrinkage is often adequate to identify the largest effects in most biological problems.

The Bayesian posterior distribution was constructed by combining the Normal likelihood with all prior distributions. A Monte Carlo Markoc Chain (MCMC) algorithm was used to obtain samples from this posterior distribution. The MCMC algorithm was a mix of Gibbs sampling updates and Metropolis–Hastings (MH) updates. The parameters *γ*, *u* and *β* (conditional on indicator variables *z*) have Normal conditional posterior distributions and were updated using Gibbs sampling steps. The *z*_i_ indicator variables to assign *β*_i_’s to mixture components were updated with MH updates, with proposals to move either to the next or to the previous mixture component with 0.5 and 0.5 probability, but when in the first component the proposal was only to move to the next, and when in the last component the proposal was only to move to the previous component. The MH probability ratio for accepting or not to move, consisted of the ratio of the likelihoods selecting the current or the new proposed mixture component for *β*_i_, and an added balancing correction when in the first or last mixture component. The vector (*π*_1,_*π*_2,_*π*_3,_*π*_4_) has a conditional posterior distribution that is Dirichlet and was updated with a Gibbs sampling step. The variance parameters $\sigma _{\rm{e}}^2$ and $\sigma _{\rm{u}}^2$ have scaled-inverse chi-square conditional posterior distributions and were also updated with a Gibbs sampling step. However, the variances in the mixture distribution needed an MH algorithm to update all variance simultaneously under the given constraint. This variance update made proposals for the new variances on a log-normal scale (obeying the needed constraints), and the MH acceptance probability is the likelihood ratio between using the current and proposed variances in the distribution of *β*.

We were interested in obtaining the variances explained by host genetics and microbial abundances on methane production, that is, var(**Zu**) and ver(**Mβ**). For var(**Zu**) we used $\sigma _{\rm{u}}^2$. However, var(**Mβ**) is less straightforward because **β** follows a mixture distribution, and we constructed the posterior distribution of var(**Mβ**) by computing this term in every cycle of the Markov chain. Finally, we obtained the posterior means, and the highest posterior density (HPD) interval (95%) for these explained variances. The Markov chain was run for 100,000 iterations, with the first 10,000 cycles discarded as burn-in. Finally, every 20th sample of the remaining 90,000 iterations were saved for the posterior analysis.

#### Detection of heritable rumen microbiota using a mixed model

We estimated the proportion of variance due to host genetic markers on OTUs collapsed at genus level or OTUs directly using a mixed model Restricted Maximum Likelihood (REML) analysis. The *p* values of the estimates of the proportion of variance explained were adjusted using multiple testing correction and a FDR using Benjamini–Yekutieli procedure [38] with adjusted *p* values smaller than 0.05 considered as significant.

We performed a principal coordinate analysis (PCoA) using a Bray–Curtis dissimilarity matrix [39] and Chao 1 [40] to examine the similarities between archaeal and bacterial rumen microbiota, using the natural log transformed and standardized matrix **M**, details see [15, 31]. PCoA and Chao 1 were used to estimate the diversity of the microbiota, corresponding to the diversity between (β) and within (α) individuals, respectively. In addition, we conducted a principal components analysis (PCA) on the CLR transformed OTU data, in order to assess microbial composition when compositionality is considered [34]. Euclidean distance was used as the dissimilarity measure used for the PCA. The following linear mixed model was fitted to estimate the proportion of variance of archaeal and bacterial rumen taxonomic groups explained by genetic markers:

$${\mathbf{y}} = 1\prime \mu + {\mathbf{Zu}} + {\mathbf{e}},$$

where **y** was a vector of abundances for one of the taxonomic groups, pre-corrected for herd, parity, days in milk and a Wilmink function on days in milk as described above. As response variable we used the first two PCoA and Chao 1 for archaeal and bacterial rumen taxa, genus level classification of archaeal and bacterial ruminal taxa, of selected archaeal and bacterial ruminal OTUs and the first to PCs (PCA analysis). 1 was a vector of ones, and *μ* was the general mean. **Z** was an incidence matrix relating phenotypes to the corresponding random polygenic effect, and **u** was a vector of random polygenic effects that follows a multivariate normal distribution $N( {0,{\mathbf{G}}\sigma _{\rm{g}}^2} )$, where **G** was the genomic relationship matrix built using 50k SNP markers following Yang et al. [41], and $\sigma _{\rm{g}}^2$ was the polygenic variance. **e** was a vector of random residuals, ${\mathbf{e}}\sim N\left( {0,{\mathbf{I}}\sigma _e^2} \right),$ where **0** was a vector of zeroes, **I** was an identity matrix, and $\sigma _{\rm{e}}^2$ was the residual variance. The genomic heritability is estimated as the ratio of polygenic variance to total phenotypic variance $h2 = \sigma _{\rm{g}}^2/\sigma _{\rm{p}}^2$. Significance of the genomic heritability was tested by using the S.E. obtained from the REML analysis.

#### Detection of association between host genetic markers and rumen microbiota using a mixed linear model

The following linear mixed model was fitted to detect significant host genetic markers affecting the composition of the microbiota, testing one host SNP at a time:

$${\mathbf{y}} = 1\prime \mu + {\mathbf{Zu}} + {\mathbf{X}}g + {\mathbf{e}},$$

where **y** was a vector of corrected phenotypes as in the previous model, i.e., the first two PCoA and Chao 1 for archaeal and bacterial rumen taxa, genus level classification of archaeal and bacterial ruminal taxa, of archaeal and bacterial ruminal OTUs explaining large proportion of variation in methane emission detected from 3.1 and the first two PCs (PCA analysis). 1 was a vector of ones, and *μ* was the general mean. **Z** was an incidence matrix relating phenotypes to the corresponding random polygenic effect, and **u** was a vector of random polygenic effects that follows a multivariate normal distribution$N( {0,{\mathbf{G}}\sigma _{\rm{g}}^2} )$, where **G** was the genomic relationship matrix built using 50k SNP markers following Yang et al. [41] and $\sigma _{\rm{g}}^2$ was the polygenic variance. **X** was a vector of allele counts (0, 1, 2); *g* was the SNP effect. **e** was a vector of random residuals, ${\mathbf{e}}\sim N\left( {0,{\mathbf{I}}\sigma _{\rm{e}}^2} \right),$ where **0** was a vector of zeroes, **I** was an identity matrix, and $\sigma _{\rm{e}}^2$ was the residual variance. This model was used to calculate the SNP effect for each SNP in the SNP chip successively. The *p* values of SNP effects were calculated using a *t* test based on the estimate and its S.E., and Bonferroni correction was applied for the *p* values of SNP effects estimates, by dividing by the number of tests (i.e., number of markers tested).

## Results

### Host additive genetics and microbiota jointly contribute to the variation in methane emission

A Bayesian mixture model was used to examine the relative contributions of host genetics and microbiota variation at the OTU level on methane emission by fitting all effects simultaneously.

The proportion of variation in methane emission explained by host genetic effects was 22% (95% HPD interval = [3%, 45%]). The proportion of variation explained by microbial OTUs after natural log transformation and standardization was 7% (95% HPD interval = [0.02%, 17%]). When fitting both host genetics and microbial OTUs, they jointly explained a total of 31% of the variation in methane emission, in which the proportion of variance explained by host genetic effects increased to 24% (95% HPD interval = [3%, 48%]) and the proportion of variance explained by microbiota remained at 7% (95% HPD interval = [0.06%, 17%]).

When treating the compositionality of microbiota data by CLR transformation, we found that the proportion of methane emission variation explained by host genetics and microbiota were 22% (95% HPD interval = [4%, 42%]) and 11% (95% HPD interval = [0.5%, 22%]) respectively, when fitting host genetics and microbiota separately. When we fit host genetics and microbiota simultaneously 11% (95% HPD interval = [0.9%, 23%]) of the methane emission variation explained by host genetics and 24% (95% HPD interval = [5%, 46%]) of the methane emission variation explained by microbiota.

**Fig. 1:**
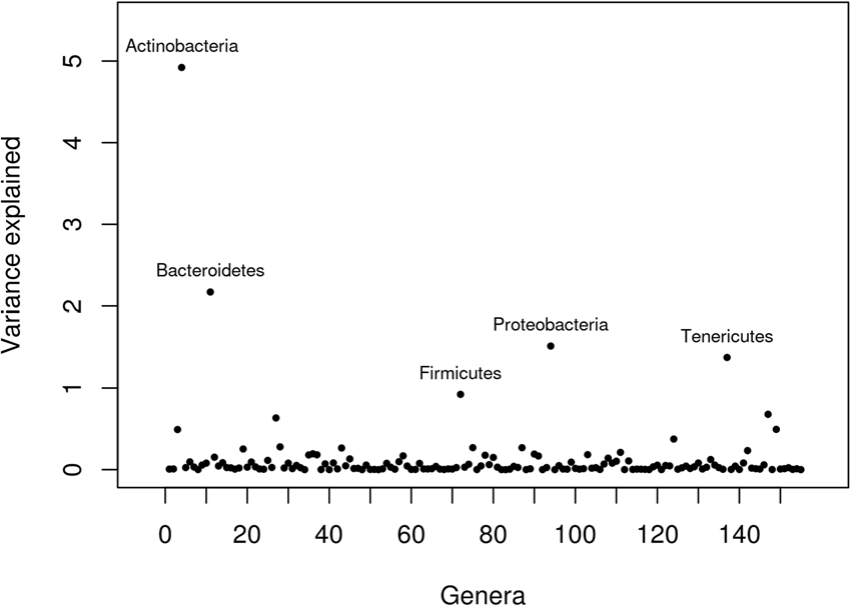
The variance explained by bacterial and archaeal genera in the variation of methane emission using Bayesian variable selection model. The *X* axis is genus numbered and the variation explained by natural log transformed and standardized bacterial and archeal genus data is in one unit of the measured methane emission.

**Fig. 2:**
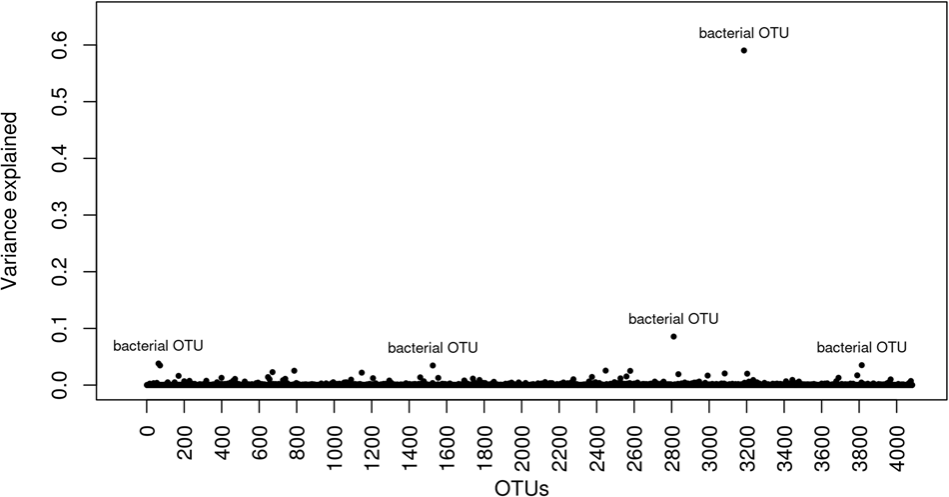
The variance explained by bacterial and archaeal OTUs in the variation of methane emission using Bayesian variable selection model. The *X* axis is OTUs numbered and the variation explained by natural log transformed and standardized bacterial and archeal OTUs data is in one unit of the measured methane emission.

We then quantified the relative contribution of each bacterial and archaeal OTU, genera abundance in the variation of methane emission from the Bayesian four-component mixture model (Table S1). Figures 1 and 2 show the exact variance explained by each genus and OTU in the variation of methane emission apart from the genetic variance. Generally, each genus and OTU explained very small amount of variation in methane emission. The largest proportion of variance explained in methane production regardless of transformation ways for microbiota data was observed for a bacterial genus (i.e., Domain: Bacteria, Phylum: Actinobacteria, Class: Coriobacteriia, Order: Coriobacteriales, Family: Coriobacteriaceae), which explained 1.6% (95% HPD interval = [0.004%, 2.4%]) of total microbiota variance, and a bacterial OTU (Phylum: Bacteroidetes, Class: Bacteroidia, Order: Bacteroidales), which explained 0.03% (95% HPD interval = [0.09%, 21%]) of the variance in methane production.

### The proportion of variance of microbiota community explained by host genetic markers using a mixed linear model

**Fig. 3:**
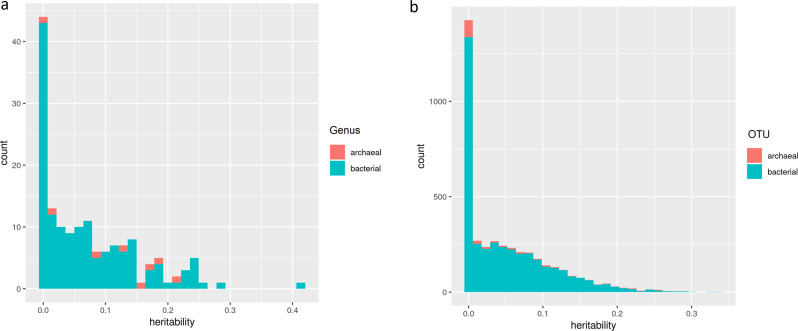
The estimates of proportion of microbiota variation explained by host genetic markers. **a** The estimates for microbiota collapsed at genus level grouped as bacterial and archaeal; **b** the estimates for OTU level grouped as bacterial and archaeal.

The composition of bacterial and archaeal communities were described in Difford et al. [15]. Here a mixed linear model was used to estimate host genomic heritability on abundances of microbial genera by REML estimates of variances. The variance proportion of microbiota community explained by genetic markers ranged from 0 to 41% with an average of 7% for abundance of bacterial genera, and from 0 to 22% with an average of 12% for abundance of archaeal genera (Fig. 3a and Table S2). Among these estimates, 16 out of 147 bacterial and 2 out of 8 archaeal genera had estimates significantly different from zero with a *p* value smaller than 0.05. The bacterial genus *Paludibacter* had the highest heritability of 41% with a standard error of 0.12. The two most significant estimates of 22% and 19% for archaeal genera were observed for the methanogenic genera *Methanobrevibactera* and *Methanosphaera*, respectively.

The variance of each bacterial and archaeal OTU explained by genetic markers was estimated. The estimates ranged from 0 to 34% with a mean of 5% for bacterial OTUs and range from 0 to 27% with a mean of 5% for archaeal OTUs (Fig. 3b and Table S2). Among the estimates, 102 out of 3897 bacterial OTUs had an estimate significantly different from zero and 15 out of 189 archaeal OTUs had an estimate significantly different from zero (*p* < 0.05). The highest estimate was observed for an unclassified bacterial OTU belonging to family Lachnospiraceae (Phylum: Firmicutes, Class: Clostridia, Order: Clostridiales, Family: Lachnospiraceae) with 33% (S.E. = 0.12, *p* = 0.004), and an archaeal OTU belonging to family Thermogymnomonas (Phylum: Euryarchaeota, Class: Thermoplasmata, Order: Thermoplasmatales, Thermoplasmatales incertae sedis, Family: Thermogymnomonas) with 26% (S.E. = 0.11, *p* = 0.01). However, after using Benjamini–Yekutieli procedure for multiple testing correction, none of the corrected *p* values is significant anymore (*p* < 0.05).

### Host genetic markers affecting the microbiota community and interacting with methane emission using a mixed model

**Fig. 4:**
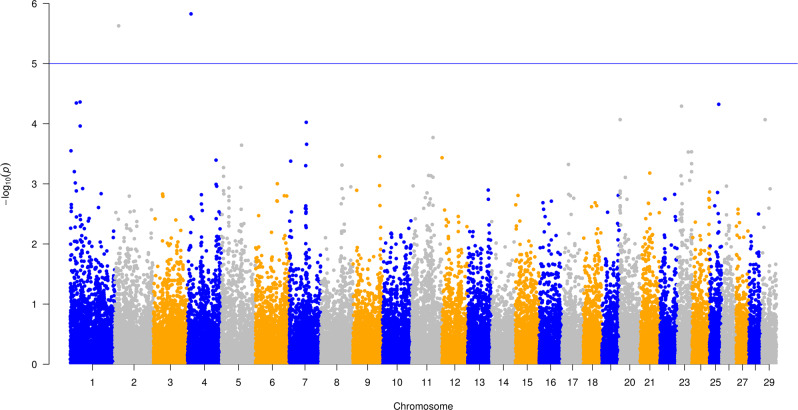
Manhattan plot of –log10(p) values for genome-wide associated markers associated with microbiota composition (α diversity). X axis is the 28 chromosomes and y axis is –log10(p) values for each marker.

PCoA was performed to characterize the variation in composition between individuals and alpha diversity estimates for variation within individuals. This information was used to associate differences in microbiota composition with host genetic markers to identify host genetic factors that cause variation between and within individuals in the composition of microbiota. A mixed linear model was used to control for background relatedness between cows, and testing the contribution of one SNP marker at a time. We found that the proportion of variance of the microbiota variation in microbiota community composition (β diversity) explained by host genetic markers were 28% (S.E. = 0.11) for the bacteria first PCoA and 22% (S.E. = 0.10) for archaeal first PCoA, respectively. This finding is comparable to previous findings in dairy and beef cattle [15, 42]. We also performed a CLR transformation of our data to account for the compositional nature and investigate the proportion of variance of the microbiota variation on the first two principal components (Table 1). The genetic markers explained 10–15% of the bacterial, and 29–38% of the archaeal, first and second principal components, respectively (Table 1). Genetic markers inside the genes such as *ABS4* and *DNAJC10* were identified to significantly associate with bacterial microbiota composition within individual (α diversity) (Fig. 4 and Table 1). However, none of the genetic markers identified significantly associate with microbiota composition between individuals (β diversity) regardless of transformation methods applied in microbiota data.

**Table 1:** The proportion of PCoA, alpha, beta diversity variation explained by host genetic markers.

Microbiota	Alpha or beta diversity	Mean	Standard deviation	Proportion of variance explained by host genetic markers	Standard error
Bacteria	Chao 1	3057.81	337.48	0.009	0.06
	PCoA1 (33.79 % explained variance)	7.25E−18	0.24	0.28	0.11
	PCoA2 (13.70 % explained variance)	1.68E−17	0.15	1.0E−6	0.05
	PC1(22.16 % explained variance)	−2.54E−15	45.81	0.15	0.09
	PC2 (5.52 % explained variance)	1.70E−15	22.86	0.10	0.07
Archaea	Chao 1	129.97	21.88	0.0038	0.06
	PCoA1 (62.17 % explained variance)	9.96E−19	0.32	0.22	0.10
	PCoA2 (14.56 % explained variance)	1.49E−18	0.16	1.0E−6	0.06
	PC1 (25.97 % explained variance)	1.55E−16	11.17	0.29	0.11
	PC2 (11.92 % explained variance)	−4.15E−16	7.57	0.38	0.09

The PCoA results come from a compositional and scaled transformation and the PC results come from a CLR transformation.

We further estimated the variance explained by genetic markers in the variation of microbial genera (Domain: Bacteria, Phylum: Actinobacteria, Class: Coriobacteriia, Order: Coriobacteriales, Family: Coriobacteriaceae) and OTU (Phylum: Bacteroidetes, Class: Bacteroidia and Order: Bacteroidales), which explained the large fraction of variance in methane emissions. The proportion of variance of this genus and OTU explained by genetic markers were very low (9% and <1%, respectively). Several highly associated markers and genes were identified for the genera and OTUs explaining the largest fraction of variance in methane emission (Tables 2 and 3). None of them, however, exceeded the significance threshold after Bonferroni correction (adjusted *p* value < 0.05).

**Table 2:** The top genetic markers associated with the bacterial OTU (i.e., Phylum: Bacteroidetes, Class: Bacteroidia and Order: Bacteroidales) explained the most variance in the variation of methane emission.

Chromosome	Position	Allele 1	Allele 2	Frequency	Effect size	Standard error	*p* value
1	118226740	A	C	0.33	−0.25	0.07	1.9e−4
1	119579301	A	G	0.32	0.26	0.07	1.4e−4
3	114797789	A	G	0.29	−0.28	0.07	1.4e−4
5	82878525	G	A	0.34	−0.27	0.07	5.7e−5
9	75484456	A	G	0.18	0.32	0.08	8.1e−5
13	9276071	G	A	0.48	0.24	0.06	2.2e−4
13	9424085	A	G	0.47	0.24	0.06	1.9e−4
15	55206099	A	G	0.47	−0.23	0.06	2.3e−4
20	7103987	A	C	0.33	0.27	0.07	1.3e−4
29	2491812	A	G	0.17	−0.32	0.09	1.9e−4

The effect sizes of SNP are in one unit of natural log transformed and standardized microbiota composition.

**Table 3:** The top genetic markers associated with the microbiota genera (i.e., Domain: Bacteria, Phylum: Actinobacteria, Class: Coriobacteriia, Order: Coriobacteriales, Family: Coriobacteriaceae) explained the most variance in the variation of methane emission.

Chromosome	Position	Allele 1	Allele 2	Frequency	Effect size	Standard error	*p* value
2	34537333	A	G	0.37	0.23	0.06	2.4e−4
3	29880508	A	G	0.22	0.29	0.07	1.2e−4
9	51243021	G	A	0.36	−0.24	0.06	1.6e−4
9	53197668	G	A	0.43	−0.23	0.06	2.2e−4
14	66907798	G	A	0.22	−0.29	0.07	1.3e−4
16	64516788	G	A	0.01	0.95	0.260	2.5e−4
17	57109193	A	G	0.38	0.27	0.07	6.1e−5
17	74925889	A	G	0.40	0.25	0.07	1.7e−4
25	37693781	A	G	0.41	−0.23	0.06	2.5e−4
25	39844749	A	G	0.21	−0.28	0.08	2.5e−4

The effect sizes of SNP are in one unit of natural log transformed and standardized microbiota OTU data.

**Fig. 5:**
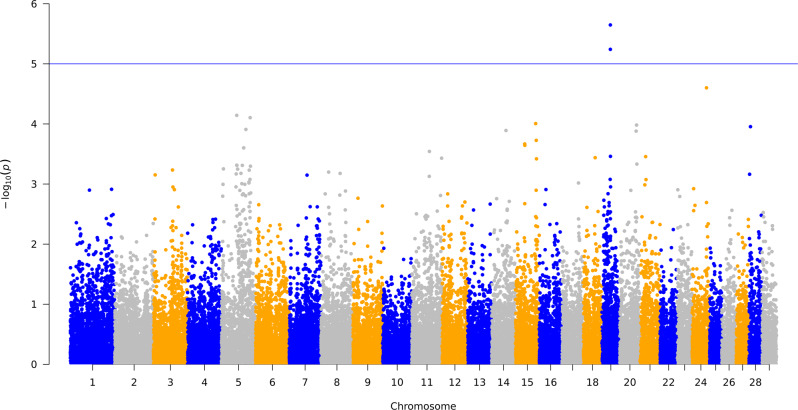
Manhattan plot of –log10(p) values for genome-wide associated markers associated with the most heritable archaeal OTU (i.e. Phylum: Euryarchaeota, Class: Thermoplasmata, Order: Thermoplasmatales, Thermoplasmatales incertae sedis, Family: Thermogymnomonas). X axis is the 28 chromosomes and y axis is –log10(p) values for each marker.

Lastly, we tested the association of genetic markers with most heritable bacterial and archaeal genus (bacterial: *Paludibacter* and archaeal: *Methanobrevibactera*) and OTUs (bacterial: Phylum: Firmicutes, Class: Clostridia, Order: Clostridiales, Family: Lachnospiraceae; archaeal: Phylum: Euryarchaeota, Class: Thermoplasmata, Order: Thermoplasmatales, Thermoplasmatales incertae sedis, Family: Thermogymnomonas). Significant genetic markers were identified for the highest proportion of variance of archaeal OTU explained by genetic markers (Fig. 5). Interesting, we found that gene *DNAH9* overlapped with markers significantly associated with this archaeal OTU.

## Discussion

This is the first use of Bayesian mixture models to estimate the proportion of variance in methane emission explained by variation in ruminal microbiota composition while jointly modeling the effects of parity, lactation stage, herd of origin, and host additive genetics in the model. Microbiota data are compositional and high-dimensional data due to the conversion to the relative taxon abundance [43–45]. A linear model approach simply fits a metagenomic relationship matrix as random effect in the model. In contrast, our Bayesian mixture model assumes that the effects of microbial groups follow a four-component mixture of normal distributions, allowing the model to classify each microbial OTU into one out of four levels of shrinkage. The Bayesian mixture model added more variance components to assign different priors for the effect sizes of microbiomes (i.e., a mixture of four normal distributions) compared with linear mixed model assuming only one normal distribution for effect sizes. The most accurate method to estimate variance component based on microbiome data is to fit all microbiotas simultaneously treating the microbiota effects as drawn from a prior distribution that matches the true distribution of microbiota effects as closely as possible. However, the true distribution of microbiota effect sizes is unknown, but a more flexible, i.e., mixture of normal distributions can approximate a wide range of distributions by varying the mixing proportions. The mixture prior on the effect sizes indeed assumes that the OTUs fall in four groups with very small/negligible effects, small, medium, and large effects, as set in the relative variances for the four components, while our Bayesian model could be developed further to treat OTU matrix to have measurement error. Our modified BayesR approach performs MCMC to create posterior distributions for parameter estimation, while estimating all parameters from the data. The used prior distributions are mostly uninformative, except for mixture proportions where the priors were chosen to indicate that more OTUs should have negligible and small effects, while only a smaller proportion can have medium and large effects. Our study reveals that a Bayesian four-component mixture models can be used to model the effects of heterogeneity, and complex relationships between microbial groups without simply assuming one simple normal distribution.

The amount of variation in methane emissions attributable to the host genetics was 24% (heritability). Under natural log transformation and standardization, the microbiota alone could explain 7% of variation when assuming the effects of OTUs follow four-mixture of normal distributions. We further compared the four-mixture model with two-mixture model. It was found that when fitting the two-mixture model, the microbiota alone explained only 6% of the total variation. The proportion of variance explained by microbiota is slightly higher using four-mixture model compared with two-mixture model. We observed that the deviance information criterion (DIC) for four-mixture model (DIC = 7048) is slightly lower than the two-mixture model (DIC = 7049). It reflects that four-mixture model is a better model and could capture more variation explained by microbiota due to its flexibility by assuming four-mixture groups of OTU effects. There are similar findings in other studies that four-mixture model had the best predictive ability compared with other models [27, 46–49]. When a two-component mixture model would be simplified further, reducing it to one component only, the model would become a regularized (ridge) regression model. As the two-component model already is slightly worse than the four-component model, we expect simplification to a ridge regression model would worsen the fit further. We also performed a sensitivity analysis within the four-component model using different priors, modifying the Dirichlet prior for the mixture proportions, and modifying the variance-ratios between the mixture components. This showed that the estimates for the microbial explained variance could change between 6 to 8.6% under different prior settings. Between priors Dir(1000,100,10,1), Dir(125,25,5,1), and Dir(1,1,1,1), the Dir(125,25,5,1) that we used had the lowest DIC. Between different setting for variance-ratios in the mixture components, we found that a more extreme setting of $\sigma _1^2 = 100\sigma _2^2 = 1000\sigma _3^2 = 10000\sigma _4^2$ obtained lower DIC and reached an explained variance of 8.6%. Overall, we concluded that the influence of the priors is small, smaller than the posterior uncertainty on these estimates, and that the priors we used struck a good middle ground with an estimated microbial contribution to explained variance of 7%. However, the performance of regression of the four-mixture model can be further improved by further optimizing priors and integrating optimized continuous weights from prior knowledge, such as prior biological knowledge of microbiota genomes or a different prior distribution such as Dirichlet multinomial mixtures [50]. When combining host genetics with the role of the microbiota, the model could jointly explain 31%. Difford et al. found that the proportion of variance of methane emission explained by fitting host pedigree matrix as a random effect was 19% and that the proportion of variance of methane emission explained by microbiota estimated in a separate model using a microbial relationship matrix was 15% [15]. In a model fitting these two effects simultaneously the variation explained by host and microbes are forced to be uncorrelated by linear mixed effect model assumptions, in this case the heritability increased to 21% and the microbiability decreased to 13%. From the relative change in explained phenotypic variation, we could conclude that the host genetics explaining variation in methane emissions was largely independent of the rumen microbiota in explaining variation in methane emissions. In the current study, we used a Bayesian mixture model with four mixed normal distributions of microbial effects assumed as opposed to a single normal distribution assumed by Difford et al. (2018) in cattle and others in pigs, poultry, and humans [51–53]. When fitting both host genetics and rumen microbes, the proportion of variance explained by host genetics increased from 22 to 24% and the proportion of variance explained by microbiota remained the same (7%). While under CLR transformation of microbiota data to account for compositionality, we observed the similar trend for the proportion of variance in methane emission variation explained by host genetics and microbiota by fitting them separately or simultaneously in the Bayesian mixture model. This suggests that the host genetics exercises the majority of its influence on ruminal methane emissions by mechanisms other than by direct host control of the composition of the ruminal microbiota.

On average, 7 and 12% variance of bacterial and archaeal taxon abundance was explained by host genetic markers. Abundance of a small proportion of bacterial and archaeal taxa had a high estimate for proportion of variance of methane emission explained by host genetic markers, implying that cows with a similar genetic background would have similar abundance of these bacterial and archaeal taxa. Compared with the estimates using pedigree information from Difford et al. [15], our estimates using genetic markers are probably more reliable, as pedigrees may be incomplete or contain errors. Compared with those authors, we also observed more significant microbial taxa with estimates significantly different from zero. Generally, estimates of proportion of variance explained by host genetic markers in microbiota abundance were low to moderate. This is consistent with a recent finding by Wallace et al. [54], who found 39 rumen microbial taxa to be heritable in 650 Holstein-Friesian cows from UK and Italy. This suggests that the host genome has a limited influence on the rumen microbiota composition. Nonetheless, the presence of highly heritable microbial taxa implies the possibility for host genetic control through the heritable microbiota for other economic traits. For example, we observed that the most heritable microbial genus was *Paludibacter*. It was suggested that *Paludibacter* is major genus propionate producer [55], while the formation of propionate was considered as a competitive pathway of hydrogen use to further produce methane [56]. This potentially provides possible ways to reduce methane emission by increasing the formation of propionate through host genetics. Further, the two methanogenic genera *Methanobrevibactera* and *Methanosphaera* with most significant estimates of proportion of microbiota variation explained by host genetic markers have been proposed to use methanol as a substrate for converting CO_2_ to methane [57]. *Methanobrevibactera* can be used to formulate H_2_ and CO_2_ to produce methane and *Methanosphaera ruminatium* also plays a role in the methanol pathway [58–60]. Therefore, our findings suggest that these microbiota might be utilized to reduce methane emission through host genetics. Notably, the sample size used in this study is comparatively modest in size for quantitative genetic analyses and increasing sample size will increase the power and precision of the estimated genetic parameters. However, this is currently the largest sample size from a single population of dairy cows to estimate the genomic heritability of rumen microbiota and thus the most reliable estimates to date.

It is important to make the distinction between rumen community composition at the OTU level or at the genus or higher taxonomical levels, as both methods have advantages and disadvantages. OTUs are defined as amplicon sequences clustered at 97% sequence similarity, i.e., de novo OTU picking [61]. In most cases the threshold of 97% sequence similarity is adequate for delineating species, however intragenic variation in the 16S rDNA sequences along with only sequencing portions of the hypervariable regions has been shown to over inflate the number of species estimated from OTUs by as much as 123% [62]. In such instances OTUs may not accurately reflect the diversity in prokaryotic species and strains and are not necessary the closest proxy to true biological diversity in a sample. Taxonomical classification of OTUs can overcome the overestimation of strains and subspecies inherent in de novo OTU picking, however this method is reliant on four publicly available databases [63], and the accuracy and reliability of these databases has not been assessed for rumen prokaryotes. This limits the discovery of OTUs to those already included in the database and cannot capture new variation. Furthermore, these databases are predominantly limited to grouping OTUs at genus level resolution. Whilst in many cases member of the same genus can have similar biological functions, some species within the same genus can have very diverse functions. For instance, the genera *Prevotella* is one of the most abundant genera in ruminants, often with 100 or more OTUs assigned [64]. Moreover, the cultured members of *Prevotella* have diverse metabolic capabilities for example hydrolysis of proteins and peptides, starch and many hemicelluloses, as well as fermentation of many amino acids and most sugars [65]. For the reasons listed above, genetic analyses were conducted at both OTU level as well as genus level.

Generally, each taxonomic group of microbes only explained a small amount of methane emission variation and jointly explained 7% of methane emission variation, which implies that the microbiota plays a relatively smaller role in variation in methane emission. However, we observed one group of microbes belonging to the family Coriobacteriaceae which explained a relatively large amount of variation in methane emissions (Domain: Bacteria, Phylum: Actinobacteria, Class: Coriobacteriia, Order: Coriobacteriales, Family: Coriobacteriaceae). Abundance of this genus of bacteria belonging to family Coriobacteriaceae has previously been found to highly negatively correlated with methane emissions in cattle and sheep [66, 67]. Our findings support this. Especially, the abundance of Coriobacteriaceae was known to be negatively correlated with CH_4_ emissions [66]. Difford et al. [15] identified several bacterial and archaeal genera, which were significantly associated with methane emission. These were *Sporobacter, Sphaerochaeta*, and *Bacteroidales*. In this study, we focused on examining the variance due to bacterial or archaeal taxa on variance in methane emission. We observed that archaea known to play important roles in methane production [68] at most explained 0.01% of variance in methane emissions. This result, however, was consistent with the observation in other studies that weak correlations between methane emission and the abundance of archaea in ruminant livestock [10, 15].

The rumen microbial community structure was examined by PCoA for the archaeal and bacterial communities as this method has been shown to produce distinct clusters called “ruminotypes” in sheep and cattle [42, 69]. However, beta diversity analysis using the PCoA approach fails to account for the compositional nature of 16S rRNA microbial data and thus we also examined microbial community structure using PCA analysis of CLR transformation [34]. We focus on exploiting host genetic markers and genes controlling the rumen community structure and important microbiota (β diversity) by use of mixed linear models. The first and second principal components (PCA) had genetic markers explaining 10–15% for bacteria and 20–38% for archaeal. Similarly, the host genetic markers explained 28 and 22% of the variance in the first principal coordinates (PCoA) of the rumen archaeal and bacterial composition. Recently, Li et al. [42] found host genetic markers to explain between 0–25% of bacterial and 0–15% of archaeal community composition (PCoA) in Beef cattle. This implies that it is possible to control microbiota composition by changing host genetics. Therefore, breeding programs can be designed to breed for cows with a specific preferred microbiota composition suited for high production efficiency or low methane emission. However, it is notable that we utilize PCoA axes as an approximation to quantifying the variance explained by the host genetic markers in the variation of PCoA axes using a mixed linear model, which ignores the non-linear curvatures of the original data space. Therefore, in our linear mixed model, host genetic markers could only capture the variance in the variation of PCoA axes in a linear relationship and the explained variance might increase when we are able to capture the nonlinearity of PCoA axes. Further, we also identified genes and genetic markers associated with the microbiota community structure. Genes *ABS4* and *DNAJC10* were found to associate with the composition bacterial community. Gene *DNAJC10* was found to associate with milk content of conjugated linoleic acid in cattle [70]. Furthermore, the microbiability of conjugated linoleic acid was found to be 33% with a standard error of 0.17 [71], which provides a strong evidence on the positive correlation of bacterial composition and milk composition. It suggests that Gene *DNAJC10* affects milk acid content through an effect on microbiota.

The gene *DNAH9* was significantly associated with the archaeal OTU (Phylum: Euryarchaeota, Class: Thermoplasmata, Order: Thermoplasmatales, Thermoplasmatales incertae sedis, Family: Thermogymnomonas) with most variance explained by host genetic markers. This archaeal OTU belongs to the recently discovered seventh order of archaea originally termed “Thermoplasmatales” [72] or “Methanoplasmatales” [73] based on their close phylogenetic relationship to nonmethanogenic Thermoplasmatales [74], which has been widely adopted across databases. However, according to Bacteriological Code the taxonomic name must be derived from the first isolate in the order, in this case Methanomassilicoccus [74]. Importantly this order are methylotrophic methanogens using H_2_ as an electron donor for reducing methylamines and methanol [72, 75]. A study on sheep showed that the composition of bacterial (i.e., PCoA) was associated with methane emission through passage rate with possible genetic mechanisms related to muscle contraction [69]. Gene *DNAH9* identified in our study encoded for axonemal beta heavy chain dynein 9 and has important biological role in muscle contraction [59]. It implies the possible genetic mechanism regulating by gene *DNAH9* in host that links this archaeal OTU and microbiota composition with impact on methane emission through passage rate. This notion is further supported by findings in beef cattle associating SNPs with *MYH3* (implicated in muscle contraction) with rumen microbial composition and feed intake [42]. Difford et al. also found numerous OTUs within this order displaying significant heritability estimates indicting these methylotrophic pathways are associated with host genetic background [15]. Gene *DNAH9* found associated with this OTU was also associated with milk conjugated linoleic acid content in cattle [70]. The gene *DNAH9* could also be a candidate gene affecting milk volatile fatty acid content through a highly heritable archaeal microbiota with a heritability of 22%. Thus, it might be possible to breed for the cows with optimized microbiota composition for improving milk-related traits and reducing methane emission through genetics. However, it is notable that there might be risk to get negative responses for other traits when selecting milk-related traits through microbiota by host genetics.

We provided biological insight by exploiting the genes affecting microbiota composition and heritable microbiota and the microbiota associated with methane emission. The genetic basis in the host for methane emission and microbiota shows that we could possibly alter microbiota composition through host genetics and it is possible to breed for cows through microbiota for better production.

## Conclusion

This study examined how much the host genetics affect the rumen microbiota and its composition in cows. Host genomes explained 0–41% of variation in the abundance of microbial groups. It reveals the host genetic effects on microbiota and the possibilities to change microbiota composition through host genetics. We are also the first to utilize a Bayesian four-component mixture approach to model the complex structure and relationship between microbiota and host genetics on methane emission. The archaeal and bacterial microbiota have been identified, which explained the most variance in microbiota variance (up to 1.6%). However, each of them explain very small amount of the methane emission variation. The identified bacterial microbiota are known to be highly correlated with methane biosynthesis, which supports the evidence for the biological role of identified microbiota in methane emission from our observations. The identification of genes *DNAH9, ABS4*, and *DNAJC10* as affecting composition of ruminal microbial communities suggests that they can serve as possible markers to modify cows’ microbiota composition. These results collectively provide an improved understanding of the genetic basis of microbiota and its composition with methane emission.

## Supplementary information

Table S1

Table S2

Description of supplementary files

## Data Availability

All microbiota sequence data is freely available at https://www.ebi.ac.uk/ena/data/view/PRJEB28065. Other related data, scripts, and instructions for method implementation are freely available at 10.5061/dryad.rjdfn2z67.
