# Supplementary material for: Bayesian modeling reveals host genetics associated with rumen microbiota jointly influence methane emission in dairy cows
Source: ISME J. 2020 May 4;14(8):2019–33. doi: 10.1038/s41396-020-0663-x (PMC7368015; doi:10.1038/s41396-020-0663-x)
Supplement: Supplementary file 3 — Description of supplementary files [file 41396_2020_663_MOESM3_ESM.pdf]

Supplementary Files

Table S1. The heritability estimates for OTUs collapsed at the genus level and for each OTU.

Table S2. The variance estimates and their 95% HPD lower and upper estimates for OTUs collapsed at the genus level and for each OTU in the variation of methane emission with host genetic fitting in the Bayesian mixture model.
